# The Effects of CoronaVac and ChAdOx1 nCoV-19 in Reducing Severe Illness in Thailand: A Retrospective Cohort Study

**DOI:** 10.3390/tropicalmed8020095

**Published:** 2023-01-31

**Authors:** Thanyarat Promlek, Tonsan Hansirisathit, Jadsada Kunno, Maytawan Thanunchai

**Affiliations:** 1 Department of Clinical Pathology, Faculty of Medicine Vajira Hospital, Navamindradhiraj University, Bangkok 10300, Thailand; 2 Department of Central Laboratory, Faculty of Medicine Vajira Hospital, Navamindradhiraj University, Bangkok 10300, Thailand; 3 Department of Research and Medical Innovation, Faculty of Medicine Vajira Hospital, Navamindradhiraj University, Bangkok 10300, Thailand; 4 Division of Clinical Microbiology, Department of Medical Technology, Faculty of Associated Medical Sciences, Chiang Mai University, Chiang Mai 50200, Thailand

**Keywords:** COVID-19, SARS-CoV-2, vaccine, CoronaVac, ChAdOx1 nCoV-19, viral load, severe disease

## Abstract

Two primary vaccines for coronavirus disease 2019 (COVID-19) have been rolled out in the mass vaccination campaign that started simultaneously with the spread of the delta variant. To explore the vaccines’ effect on reducing viral load and disease severity, we conducted a retrospective cohort study in Thai patients aged ≥18 years who were confirmed COVID-19 positive by RT-PCR. Compared to unvaccinated patients, Ct values and the number of severe cases among vaccine regimens were analyzed. Ct values of vaccinated patients were not significantly different from unvaccinated patients, despite an increase of Ct values in a booster dose. The adjusted odd ratio for prevention of delta-related severe diseases was 0.47, 95% CI: 0.30–0.76 and 0.06, 95% CI: 0.01–0.45 after receiving one dose and two doses, respectively. No severe illness was found in booster-vaccinated individuals. Focusing on the vaccine types, one dose of ChAdOx1 nCoV-19 gave significant protection, whereas one dose of CoronaVac did not (0.49, 95% CI: 0.30–0.79, *p* = 0.003 vs. 0.28, 95% CI: 0.04–2.16, *p* = 0.223). Two-dose vaccination showed robust protective effects in all subpopulations regardless of vaccine type. Vaccinations with two primary vaccines could not reduce viral load in patients with COVID-19, but could prevent severe illness.

## 1. Introduction

Coronavirus disease 2019 (COVID-19), caused by severe acute respiratory syndrome coronavirus 2 (SARS-CoV-2), is entering its third year of being a pandemic and is still far from over. The emergence of variants of concern including alpha, beta, gamma, delta, and up to the recent omicron gives rise to a substantial increase in cumulative infections and deaths [1]. Vaccines are hoped to slow down the spread of the virus and alter the COVID-19 pandemic to an endemic, which eventually brings back normal life. Thailand is currently experiencing the fifth and the latest COVID-19 wave driven by the omicron variant, which is the most contagious causing the highest record of daily confirmed cases but less severe diseases compared with the delta variant in the previous wave (July–December 2021) [2]. However, the delta surge was considered the worst COVID-19 wave in Thailand because of the rising number of hospitalized and deceased patients amid the low vaccination rates and shortages of vaccine supplies [3].

COVID-19 vaccinations in Thailand have been rolled out since March 2021, and to achieve herd immunity, a mass immunization campaign was later started in June 2021 [4]. The vaccine allocation was prioritized by high-risk populations and living areas. Frontline healthcare workers (HCWs), people with underlying chronic conditions, and people aged ≥60 years were prioritized target populations for vaccination [5]. Given the high number of COVID-19 cases in Bangkok, the capital city of Thailand, it was classified as a red zone and considered a top priority for vaccination [6,7].

Two vaccine platforms were available in the earlier phase. From March to May 2021, CoronaVac (Sinovac; SV), an inactivated whole virus vaccine, was mainly offered for frontline medical personnel and recommended at a four-week interval between the first and second doses. In June 2021, ChAdOx1 nCoV-19 (AZD1222, Oxford/AstraZeneca; AZ), a replication-deficient adenovirus vector vaccine, was available for the majority of the Thai population, and it was administered up to 12 weeks apart for full vaccination [8]. In addition, BNT162b2 mRNA vaccines (Pfizer-BioNTech, New York, NY, USA), which were of limited supply at the early phase, were imported after a few months of mass immunization and implemented to be a booster vaccination. CoronaVac and ChAdOx1 nCoV-19 vaccines have been demonstrated to be highly protective against symptomatic disease, hospitalization, severe disease, and COVID-19-related death caused by other variants circulating before the emergence of the delta variant. The vaccine effectiveness (VE) of the two-dose CoronaVac were 50%–65% and 80%–100% for the prevention of symptomatic COVID-19 and severe COVID-19, respectively, caused by the gamma and alpha variants [9,10]. Similarly, full vaccination with ChAdOx1 nCoV-19 provided protection against symptomatic COVID-19 and hospitalization among patients infected with the gamma and zeta variants with VE of 60% and 95%, respectively [11].

The delta variant emerged carrying crucial mutations on spike protein, enabling the virus to increase the transmissibility and escape the immune response. It became globally dominant with higher numbers of daily cases than previous variants. Since the mass immunization campaign has started concurrently with the circulation of the delta variant in Thailand, this interests us to estimate the effects of CoronaVac and ChAdOx1 nCoV-19 vaccines for decreasing viral load and severe COVID-19. This study was conducted during the period from July to December 2021 in Vajira Hospital, a teaching university hospital that has rolled out COVID-19 vaccines for all high-risk groups and general populations living in the Bangkok Metropolitan area.

This study aimed to explore the effects of one-dose and two-dose CoronaVac and ChAdOx1 nCoV-19 regimens and a booster vaccination (two-dose CoronaVac plus ChAdOx1 nCoV-19 or BNT162b2) for reducing viral load, in which we used Ct values as a proxy for the quantity of virus, and for preventing severe COVID-19 stratified by sex, age, and pre-existing comorbidities.

## 2. Materials and Methods

### 2.1. Study Design

This retrospective cohort study aimed to estimate the effects of the COVID-19 vaccine against the delta variant in Thailand. Data were obtained from the Vajira Hospital, Navamindradhiraj University, Thailand. The study protocol was approved by the Institutional Review Board of the Faculty of Medicine of Vajira Hospital, Navamindradhiraj University (COA 048/2565) (Study code 297/64 E).

### 2.2. Population

The participants were patients aged ≥18 years who tested positive for SARS-CoV-2 infection confirmed through reverse-transcriptase polymerase transcription (RT-PCR) at Vajira Hospital between 27 July and 31 December 2021. This period covered the fourth COVID-19 wave in Thailand, in which delta variants predominated [12]. Vajira Hospital is a 900-bed tertiary medical center and a Bangkok metropolitan hospital. During the fourth wave of the COVID-19 pandemic, Vajira Hospital established 15 COVID-19 wards, two field hospitals, and a telemedicine system to serve the population in Bangkok, the capital city of Thailand. Patients who were previously diagnosed with COVID-19 or patients with incomplete data regarding the variable of interest were excluded.

### 2.3. Study Protocol and Definitions

The demographic characteristics, comorbidities, patient symptoms, RT-PCR results, treatment, immunization, and history of COVID-19 infection of each patient were retrieved from electronic medical records, physical records, and electronic public health information systems. The patient’s condition was documented from the first positive SARS-CoV-2 test until hospital discharge. Severe disease is defined as the presence of dyspnea, with SpO2 < 96% or a drop in SpO2 ≥ 3% after exercise. Vaccination status was defined on the date of testing positive on RT-PCR as follows: (1) The unvaccinated group included patients who did not receive COVID-19 vaccination or received only one vaccine dose in <14 days. (2) The one-dose group included patients who received their first dose of CoronaVac or ChAdOx1 nCoV-19 in ≥14 days. (3) The two-dose group included patients who were fully vaccinated with CoronaVac or ChAdOx1 nCoV-19 for ≥14 days after the second dose. (4) The three-dose group included patients who received BNT162b2 or ChAdOx1 nCoV-19 as a third dose for >7 days [13,14]. In this study, the underlying conditions that increased risks for severe COVID-19 outcomes followed the CDC’s updated list based on evidence in all published reports, including obesity (body mass index >27 kg/m^2^), asthma, bronchiectasis, tuberculosis, cancer, cerebrovascular disease, chronic kidney disease, chronic liver disease, COPD, diabetes mellitus, and cardiovascular disease [15].

All COVID-19 cases were confirmed by RT-PCR, which was performed at the Biomolecular Laboratory, Vajira Hospital. Briefly, nasopharyngeal swabs were collected and placed in viral transport media (VTM). RNA was extracted from aliquots of VTM on a Zybio Nucleic Acid Extraction Kit platform (Zybio Inc., China). The detection of *ORF1ab* and *N* genes of SARS-CoV-2 and the internal control was performed with RT-PCR using the Sansure Novel coronavirus (2019-nCoV) Nucleic Acid Diagnostic Kit (PCR-fluorescence Probing; Sansure Biotech, China). The Slan 96P Real-Time PCR System (Sansure Biotech) was used for amplification. The result was analyzed using ABI 7500 software. Only the first RT-PCR positive test for each patient was used in the analysis.

The effect of vaccination on the delta variant was estimated based on two outcomes. The primary outcome was the risk reduction of severe illness. The secondary outcome was a decrease in viral load. Ct values of RT-PCR represent the number of PCR cycles needed to detect SARS-CoV-2. A lower Ct value indicates a higher viral load. Thus, we compared the Ct value between the vaccinated group and the unvaccinated group.

### 2.4. Statistical Analysis

Statistical analysis was performed using IBM SPSS Statistics for Windows version 28 (IBM Corp., Armonk, NY, USA). Categorical variables were presented as frequency and percentage. Continuous variables were demonstrated as median and standard division. The Chi-square test, Fisher’s exact test, or ANOVA with the Bonferroni test were used for comparison as appropriate. Vaccines related to severe disease were analyzed by univariate and multivariate regression analysis and the relationship was presented with an odds ratio (ORs) and 95% confidence intervals (95% CIs). Subgroup analysis was stratified by age, sex, and comorbidities. The significance level was set at 0.05.

### 2.5. Ethics Committee Approval

This study was approved by the Institutional Review Board of the Faculty of Medicine Vajira Hospital, Navamindradhiraj University (Study code 297/64 E; COA 048/2565). The review board waived the requirement for individual informed consent.

## 3. Results

During the study period, 1149 patients aged ≥18 years tested positive for COVID-19. A total of 98 patients were excluded because of incomplete information or prior infection. Finally, 1051 patients met the inclusion criteria. Of the 1051 patients, 526 (50%) were unvaccinated and 525 (50%) were vaccinated. Among the 525 vaccinated cases, 382 (72.8%) received one-dose vaccination (37 for CoronaVac and 345 for ChAdOx1 nCoV-19), 131 (25.0%) received two-dose vaccination (100 for CoronaVac and 31 for ChAdOx1 nCoV-19), and only 12 (2.2%) received a booster injection (two-dose CoronaVac supplemented with BNT162b2 or ChAdOx1 nCoV-19). The characteristics of the vaccinated and unvaccinated groups are shown in Table 1. Gender and the presence of pre-existing comorbidities were comparable in both vaccination statuses. The unvaccinated group was younger than the vaccinated one. The *N*-gene Ct value was compared among unvaccinated and vaccination subgroups to investigate the effect of the vaccines on decreasing viral load. The result showed that one-dose and two-dose vaccinations had mean Ct values of 22.25 ± 6.95 and 21.46 ± 6.51, respectively, which were similar to unvaccinated (22.18 ± 6.61). Although the mean Ct value of three-dose (25.58 ± 9.97) was 3.40 (*p* = 0.513) cycles higher than unvaccinated, the difference was not significant (Table 2).

The Ct value of all RT-PCR-positive cases was stratified by vaccination status. The difference between the unvaccinated and vaccinated group was compared using one-way ANOVA with the Bonferroni test.

Among 1051 patients with COVID-19, 102 (9.7%) developed severe disease, of whom 66 (64.7%) were unvaccinated and 35 (34.3%) and one (1.0%) received one-dose and two-dose vaccination, respectively. No severe disease occurred in the three-dose vaccination. Of 102 severe cases, there were 11 deaths; nine were unvaccinated and two were one-dose vaccinated with ChAdOx1 nCoV-19 (data not shown). We estimated the protective effect of primary vaccines on severe COVID-19 compared to the unvaccinated group. One-dose vaccination reduced the risk of severe disease, but the effect was not statistically significant (OR: 0.70, 95% CI: 0.46–1.08, *p* = 0.111). The risk of severe disease was considerably reduced in the two-dose vaccination (OR: 0.05, 95% CI: 0.01–0.39, *p* = 0.004). After adjusting for sex, age, and comorbidities, both one-dose and two-dose vaccinations significantly decreased the risk of severe disease (OR_adj_: 0.47, 95% CI: 0.30–0.76, *p* = 0.002, OR_adj_: 0.06, 95% CI: 0.01–0.45, *p =* 0.006, and 0, respectively) (Table 3). Since there was no severe illness from a booster dose and the odds of reduced risk of two-dose vaccination were higher than that of one-dose vaccination, these results suggested that the level of protection of severe disease increased with the number of vaccine doses. However, the result of a booster vaccination might be overestimated by the small sample size (12 cases). To analyze the association of each vaccine regimen with severe COVID-19, we found that one-dose ChAdOx1 nCoV-19 vaccination had significantly decreased the risk of severe disease (OR_adj_: 0.49, 95% CI: 0.30–0.79, *p* = 0.003), while one-dose CoronaVac vaccination showed no significant effect. Two-dose of ChAdOx1 nCoV-19 greatly reduced the odds of progressing to severe disease (OR_adj_: 0.15, 95% CI: 0.02–1.18, *p* = 0.072), although the *p*-value was slightly higher than 0.05 (Table 3). No severe outcome was observed in patients vaccinated with two-dose CoronaVac and booster vaccinations. For subgroup analysis, after adjusting confounding factors, one-dose vaccination had significantly reduced the risk of severe disease in males, elderly patients aged over 60 years old, and patients with or without comorbidities. Two-dose vaccination showed a greater protective effect in females, the elderly, and people with comorbidities. Moreover, no severe illness was found in those males, people aged 18–59 years, and patients without underlying diseases who received two-dose vaccination (Table 4). Our findings suggest that CoronaVac and ChAdOx1 nCoV-19, which were used as primary vaccine series in the mass vaccination program, significantly reduced the severe outcomes during the period when the delta variant was circulating in Thailand and two-dose as well as booster vaccinations better offered significant protection against severe COVID-19 in all population subgroups.

## 4. Discussion

Several vaccine types have been manufactured and globally distributed one year after the COVID-19 pandemic to achieve herd immunity. Thailand encountered vaccine supply shortages, and vaccine hesitancy delayed the launching of mass vaccination campaigns. CoronaVac was the primary vaccine series for frontline healthcare personnel and people aged 18–59 years, while ChAdOx1 nCoV-19 was mainly offered for people aged ≥60 years, people with underlying diseases, and the general population. By the time these two vaccines were rolled out, the delta variant was circulating in Thailand and became predominant in the fourth wave in Thailand (July–December 2021) [3,16].

In this study, we investigated whether vaccination with the two primary vaccine series could reduce viral load and disease severity against the delta variant. Our findings demonstrated that all vaccine regimens could not decrease the viral load in the vaccinated group when compared with the unvaccinated group. Little is known about whether CoronaVac and ChAdOx1 nCoV-19 could reduce delta viral loads in vaccinated individuals. A number of studies highlighted the variable impact of mRNA vaccines against the delta variant. Levine-Tiefenbrun M. et al. [17] and Abu-Raddar L.J. et al. [18] reported that full vaccination with mRNA vaccines effectively reduced viral loads, suggesting a decreased infectiousness in vaccinated individuals. On the contrary, several studies [19,20,21,22] did not find a significant difference in Ct values between the vaccinated and unvaccinated groups. It should be noted that the Ct values did not entirely reflect the infectiousness since both incomplete and complete virus particles could be quantified by real-time RT-PCR. However, recent publications reported that two-dose vaccination significantly reduces infectious virus particles and contributes to limiting viral transmission [23,24]. Although in this study, the Ct-values were not different among vaccination subgroups, the increased Ct-values were observed only in a booster vaccination, implying that receiving a third dose of vaccines has the potential to reduce the quantity of virus in the respiratory tract. Our findings supported the importance of a booster vaccination in combination with strict preventive measures during the delta surge.

The protective effect of both CoronaVac or ChAdOx1 nCoV-19 against severe disease caused by the delta variant was estimated. The risk of severe outcomes was expressed as an adjusted odd ratio. One-dose vaccination sufficiently reduced the risk of severe disease. Higher risk reduction was apparent in two-dose vaccination and no severe cases were found after receiving a booster vaccination. The level of protection is likely to be vaccine-dose dependent. This was consistent with another study from Thailand, which demonstrated that the protective effects against delta variant-related severe disease increased with the number of vaccination doses [16]. Focusing on vaccine types, one dose of ChAdOx1 nCoV-19 significantly reduced the risk for severe illness, while one dose of CoronaVac did not. The distinct immunogenicity between CoronaVac and ChAdOx1 nCoV-19 better supported our findings. The level of IgG and neutralizing antibodies against the delta variant of ChAdOx1 nCoV-19 recipients were higher than CoronaVac recipients after partial and full vaccinations [25]. The immunity level of CoronaVac has increased after receiving the second dose; Angkasekwinai N. et al. reported ten times increase in antibody levels in those who received a second dose of CoronaVac [25]. It is possible that an increase in immunogenicity after the second dose confers protection since multiple countries that used CoronaVac as a primary vaccine addressed great protection against severe disease caused by VOCs in fully vaccinated individuals [26,27,28,29]. Two doses of ChAdOx1 nCoV-19 could lower the risk of progressing to severe outcomes. The low number of fully vaccinated patients included during the study could be explained by a long interval between the first and second doses of ChAdOx1 nCoV-19 (12 weeks). Although the true protective effects of the vaccine might be overestimated by a small sample size, the risk reduction of two-dose ChAdOx1 nCoV-19 in this study was comparable to real-world effectiveness of ChAdOx1 nCoV-19 which had 80–100% protection against severe diseases and deaths [30,31,32].

COVID-19 is much more severe in people aged 60 and older and people with underlying conditions, thus these two groups were prioritized for COVID-19 vaccines [33]. One-dose vaccination had significant protective effects against severe illness in older people, and people with medical conditions. According to the deployment of ChAdOx1 nCoV-19 for elderly and immunocompromised individuals in Thailand, the observed protective effects of single-dose vaccination were possibly inferred from ChAdOx1 nCoV-19’s efficacy. The risk reduction for vulnerable groups was improved after two-dose vaccination. For people aged 18–59 years and people without pre-existing conditions, two-dose vaccination regardless of vaccine type had highly protective effects against delta-related severe illness. Males were found to have a lower risk compared to females after receiving first-dose and second-dose vaccinations. Still, it is currently difficult to conclude the sex disparity in the efficacy of ChAdOx1 nCoV-19 and CoronaVac. Sex-based vaccine effectiveness remains a knowledge gap.

There were several limitations in this study. First, the patients’ information was from a single hospital; thus, the results may not be generalizable to other settings or populations. Second, we estimated the protective effect against severe disease only in COVID-19-confirmed cases, but not in those who tested negative. Therefore, the results might not reflect absolute protection for the whole population. Third, based on the delta prevalence report in Thailand, the percentage of the delta variant in July–December 2021 was 64–100% [34]; we assumed that the delta variant was the dominant strain in the region during our study period without performing sequence analysis.

## 5. Conclusions

Our findings demonstrate that the delta viral load was similar among unvaccinated and vaccinated subgroups, but the viral load decreased after booster vaccination with no significant difference. One dose of ChAdOx1 nCoV-19 provided greater protection against severe disease than one dose of CoronaVac. Receiving a two-dose regimen of either CoronaVac or ChAdOx1 nCoV-19 and a vaccine booster exhibited highly protective effects in all subpopulations.

## Figures and Tables

**Table 1:** Characteristics of COVID-19-positive cases in this study.

Characteristics	COVID-19			
	Total (1051)	Unvaccinated (*n* = 526)	Vaccinated (*n* = 525)	*p*-Value
**Gender *N* (%)**				
Female	628 (59.8)	304 (57.8)	324 (61.7)	0.195
Male	423 (40.2)	222 (42.2)	201 (38.3)	
**Age *N* (%)**				
18–59 years	778 (74.0)	419 (79.7)	359 (68.4)	<0.001
≥60 years	273 (26.0)	107 (20.3)	166 (31.6)	
**Comorbidities *N* (%)**				
Non-disease	856 (81.4)	438 (83.3)	418 (79.6)	0.128
≥1 disease	195 (18.6)	88 (16.7)	107 (20.4)	
**Vaccinated status *N* (%)**				
Unvaccinated	526 (50.0)	526 (100)	-	-
One dose	382 (36.3)	-	382 (72.8)	
Two doses	131 (12.5)	-	131 (25.0)	
Three doses	12 (1.1)	-	12 (2.2)	
**Vaccine type *N* (%)**				
SV1	37 (7.0)	-	37 (7.0)	-
AZ1	345 (65.7)	-	345 (65.7)	
SV2	100 (19.0)	-	100 (19.0)	
AZ2	31 (5.9)	-	31 (5.9)	
Boost	12 (2.3)	-	12 (2.3)	

Note: SV1 = one-dose CoronaVac, AZ1 = one-dose ChAdOx1 nCoV-19, SV2 = two-dose CoronaVac, AZ2 = two-dose ChAdOx1 nCoV-19, Boost = two-dose CoronaVac plus with one-dose ChAdOx1 nCoV-19 or BNT162b2.

**Table 2:** The impact of vaccines on reducing viral load.

Vaccination Status		*N*-Gene Ct Value			
	*N*	Mean (95% CI)	SD	Difference in Mean (95% CI)	*p*-Value
Unvaccinated	526	22.18 (21.611–22.74)	6.61	ref	
One dose	382	22.25 (21.56–22.95)	6.95	−0.08 (−1.28–1.13)	1.000
Two doses	131	21.46 (20.34–22.59)	6.51	0.72 (−1.13–2.57)	1.000
Three doses	12	5.58 (21.75–22.57)	9.97	−3.40 (−8.62–1.82)	0.513

**Table 3:** Vaccine regimens associated with reducing severe disease.

Variables	Non-Severe *N* = 949	Severe *N* = 102	Crude OR (95%CI)	*p*-Value	Adjusted OR (95%CI)	*p*-Value
**Vaccination status**						
Unvaccinated	460	66	1		1	
One dose	347	35	0.70 (0.46–1.08)	0.111	0.47 (0.30–0.76)	0.002
Two doses	130		0.05 (0.01–0.39)	0.004	0.06 (0.01–0.45)	0.006
Three doses	12		No event			-
**Vaccination type**						
Unvaccinated	460	66	1		1	
SV1	36	1	0.19 (0.03–1.44)	0.108	0.28 (0.04–2.16)	0.223
SV2	100	0	No event			-
AZ1	311	34	0.76 (0.49–1.18)	0.224	0.49 (0.30–0.79)	0.003
AZ2	30	1	0.23 (0.03–1.73)	0.154	0.15 (0.02–1.18)	0.072
Boost	12	0	No event			-

OR, odd ratio; CI, confidence interval; OR_adj_, adjusted odd ratio. SV1 = one-dose CoronaVac, AZ1= one-dose ChAdOx1 nCoV-19, SV2 = two-dose CoronaVac, AZ2 = two-dose ChAdOx1 nCoV-19, Boost = two-dose CoronaVac plus with one-dose ChAdOx1 nCoV-19 or BNT162b2.

**Table 4:** Estimate the effect of vaccines against severe disease stratified by sex, age, and underlying medical condition.

	Unvaccinated (Ref.)	1 Dose Vaccinated					2 Doses Vaccinated				
	*N*	*N*	OR (95%CI)	*p*-Value	OR_adj_ (95%CI)	*p*-Value	*N*	OR (95%CI)	*p*-Value	OR_adj_ (95%CI)	*p*-Value
**Female**											
Non-severe	274	213	1		1		86	1		1	
Severe	30	21	0.91 (0.51–1.63)	0.750	0.60 (0.32–1.13)	0.112	1	0.11 (0.01–0.79)	0.029	0.14 (0.02–1.05)	0.056
**Male**											
Non-severe	186	136	1		1		44	1		1	
Severe	36	14	0.53 (0.28–1.03)	0.059	0.36 (0.18–0.73)	0.005	0	0	-	0	-
**Age 18–59**											
Non-severe	390	223	1		1		111	1		1	
Severe	29	14	0.84 (0.44–1.63)	0.615	0.80 (0.41–1.56)	0.802	0	0	-	0	-
**Age ≥ 60**											
Non-severe	70	124	1		1		19	1		1	
Severe	37	21	0.32 (0.17–0.59)	<0.001	0.31 (0.17–0.58)	<0.001	1	0.10 (0.01–0.77)	0.027	0.12 (0.02–0.92)	0.042
**With underlying medical conditions**											
Non-severe	398	267	1		1		120	1		1	
Severe	40	18	0.67 (0.38–1.20)	0.175	0.50 (0.27–0.92)	0.025	1	0.08 (0.1–0.61)	0.014	0.08 (0.01–0.60)	0.014
**Without underlying medical conditions**											
Non-severe	62	80	1		1		10	1		1	
Severe	26	17	0.51 (0.25–1.02)	0.055	0.42 (0.20–0.87)	0.020	0	0	-	0	-

OR, odd ratio; CI, confidence interval; OR_adj_, adjusted odd ratio.

## Data Availability

The data presented in this study are not publicly available due to confidentiality, but are available on reasonable request.
